# What Lies Behind the Cannonball Pulmonary Metastases: Hodgkin's Lymphoma?

**DOI:** 10.7759/cureus.24351

**Published:** 2022-04-21

**Authors:** Hiba Ramdani, Ghanam Ayad, Othman Moueqqit, Abdelilah Lahmar, Samia Malki, Amal Bennani, Imane Kamaoui, Noufissa Benajiba

**Affiliations:** 1 Medicine, Faculty of Medicine and Pharmacy/Mohammed VI University Hospital, Oujda, MAR; 2 Pediatric Hematology, Faculty of Medicine and Pharmacy/Mohammed VI University Hospital, Oujda, MAR; 3 General Medicine, Faculty of Medicine and Pharmacy/Mohammed VI University Hospital, Oujda, MAR; 4 Anatomopathology, Faculty of Medicine and Pharmacy/Mohammed VI University Hospital, Oujda, MAR; 5 Radiology, Faculty of Medicine and Pharmacy/Mohammed VI University Hospital, Oujda, MAR

**Keywords:** metastasis, hodgkin's lymphoma, children, pediatric hodgkin lymphoma, tuberculosis, pulmonary metastases, cannonball

## Abstract

The cannonball pulmonary appearance is hematogenous dissemination of various primary tumors but rarely a Hodgkin's lymphoma, a disease that most commonly manifests with lymphadenopathy, often affecting the mediastinum and supraclavicular or cervical lymph nodes. To date, to the best of our knowledge, no case has been reported where the investigation of a cannonball pulmonary appearance led to the diagnosis of Hodgkin's lymphoma. Hence, in our case report, we attempt to highlight the uncommon presentation of this disease in a 14-year-old girl who initially presented with dyspnea before her chest x-ray revealed a cannonball pulmonary appearance, which was later linked with Hodgkin's lymphoma after performing a biopsy of her axillary node.

## Introduction

Hodgkin's lymphoma (HL) is one of the most common lymphoid malignancies with an annual incidence of three cases per 100,000 individuals in the western world [1]. The disease is unique in its bimodal age distribution, as most diagnosed patients are either between the ages of 15 and 35 years or above 55 years [2]. It is also the most diagnosed cancer among adolescents and young adults as it accounts for half of the lymphoma cases in this age group [2,3]. Its etiology is still unknown but risk factors such as prior Epstein Barr virus (EBV) infection and immunocompromising conditions (organ transplantation or HIV infection) are widely involved [3]. Two histological types of HL are defined according to the WHO 2008 classification: the nodular lymphocyte-predominant and the classical Hodgkin's lymphoma (CHL). CHL includes four entities: nodular sclerosis, lymphocyte depletion, mixed cellularity, and lymphocyte-rich [4]. The disease remains curable with an excellent prognosis among pediatric patients as the five-year survival rate is estimated at 98% after chemotherapy alone or coupled with radiotherapy [3].

Malignancies, in general, can be underdiagnosed among adolescents and young adults as they are less expected at this age compared to older age groups, specifically if manifested with uncommon clinical presentations. HL is often revealed by asymptomatic superficial lymphadenopathies, as the cervical, supraclavicular, and mediastinal lymph nodes are the most common sites [1]. Unexplained recurrent fever, night sweats, and weight loss are also very common during the diagnostic workup [3]. However, other uncommon circumstances could make the diagnosis more challenging and less envisioned. Hence, we intend, through our case, to share our diagnostic attitude facing a dyspneic 14-years-old girl with a cannonball pulmonary appearance in her chest x-ray, which was later found to be secondary to HL.

## Case presentation

We report the case of a 14-year-old girl with no past medical or surgical history who presented to the emergency room with complaints of dyspnea associated with a dry cough, asthenia, and unquantified weight loss of one-month duration. The patient had no dysphagia, hemoptysis, or night sweating. She also denied any contact with tuberculosis (TB) or coronavirus disease 2019 (COVID-19) patients. Prior to her admission, she received broad-spectrum antibiotics in another hospital as their diagnostic work-up revealed no precise cause before she was referred to our hospital for further investigation after a worsening of her condition.

**Figure 1:**
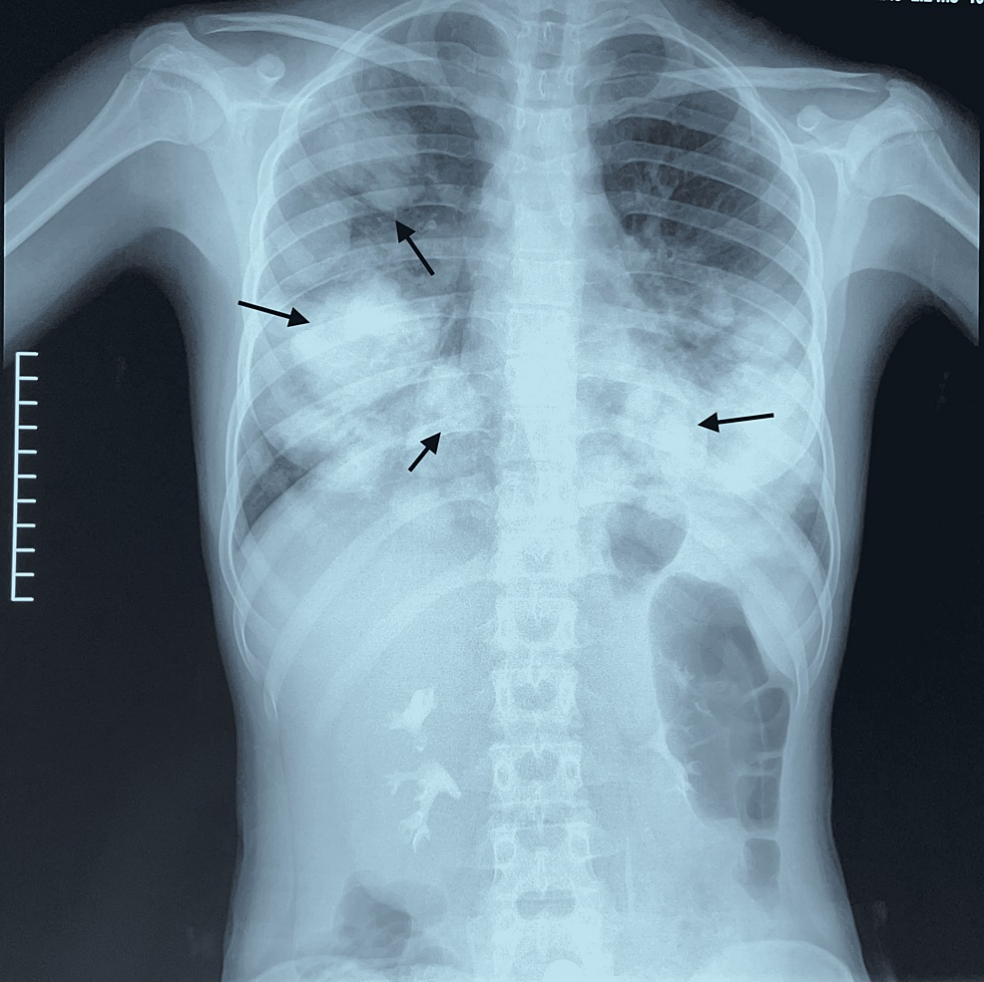
Chest x-rays of the patient, a 14-year-old girl, showed bilateral rounded nodules with cannonball appearance (arrows), highly evocative of pulmonary metastases.

Upon admission, the patient was alert, well oriented in time and space, with a Glasgow Coma Scale (GCS) of 15/15, febrile at 38.3°C with a heart rate of 113 beats/minute, blood pressure of 124/75 mmHg, respiratory rate of 25/min, and oxygen saturation of 86% on room air. On physical examination, we noted decreased vocal fremitus and breath sound evoking a condensation pulmonary syndrome, with associated bilateral, lenticular lymphadenopathies in the inguinal and submandibular areas. The rest of the physical examination revealed no abnormalities. A chest x-ray was performed and showed multiple and bilateral pulmonary nodules of various sizes, also known as “cannonball appearance” (Figure 1).

Initial complete blood count revealed microcytic hypochromic anemia with hemoglobin at 8,8 g/dL, mean corpuscular hemoglobin concentration (MCHC) at 29%, a mean corpuscular volume at 62 fL, a slight increase in white cells count (11020 /mm3) and neutrophils (9130/mm3), and a high level of lactate dehydrogenase (LDH) (2972 IU/L), while the liver and kidneys functions tests were normal. Serologies of hepatitis C virus (HCV), hepatitis B virus (HBV), HIV, and EBV were negative.

**Figure 2:**
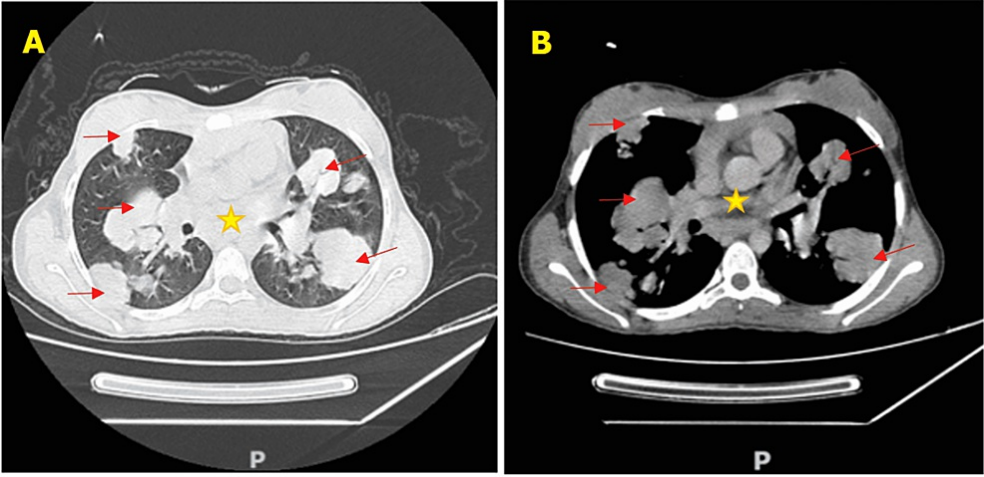
Axial chest CT scan: (A) lung window and (B) mediastinal window demonstrating bilateral and asymmetric pulmonary intra-parenchymal condensations (arrows) with mediastinal adenopathies (star).

**Figure 3:**
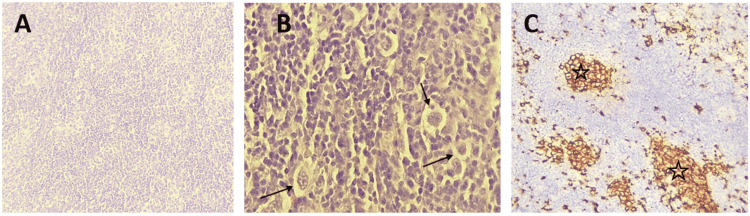
An axillary node biopsy: A. Microphotography showing a lymph node parenchyma whose architecture has been erased and replaced by a tumor proliferation arranged in diffuse layers (H&E, x100); B. Tumor cells are large, nucleated, and sometimes binucleated (H&E,x400) (arrows); C. Tumor cells express CD30 (stars). H&E: hematoxylin and eosin stain

High suspicion of an underlying malignancy with pulmonary metastasis was suggested and a CT scan of the thorax, abdomen, and pelvis with and without injection of contrast was obtained (Figure 2) and showed bilateral, large, well-circumscribed, and asymmetric pulmonary nodules with mediastinal lymphadenopathy. A CT-guided axillary node biopsy was performed, which confirmed the diagnosis of nodular sclerosis classic Hodgkin's lymphoma (NSCHL) (Figure 3).

**Figure 4:**
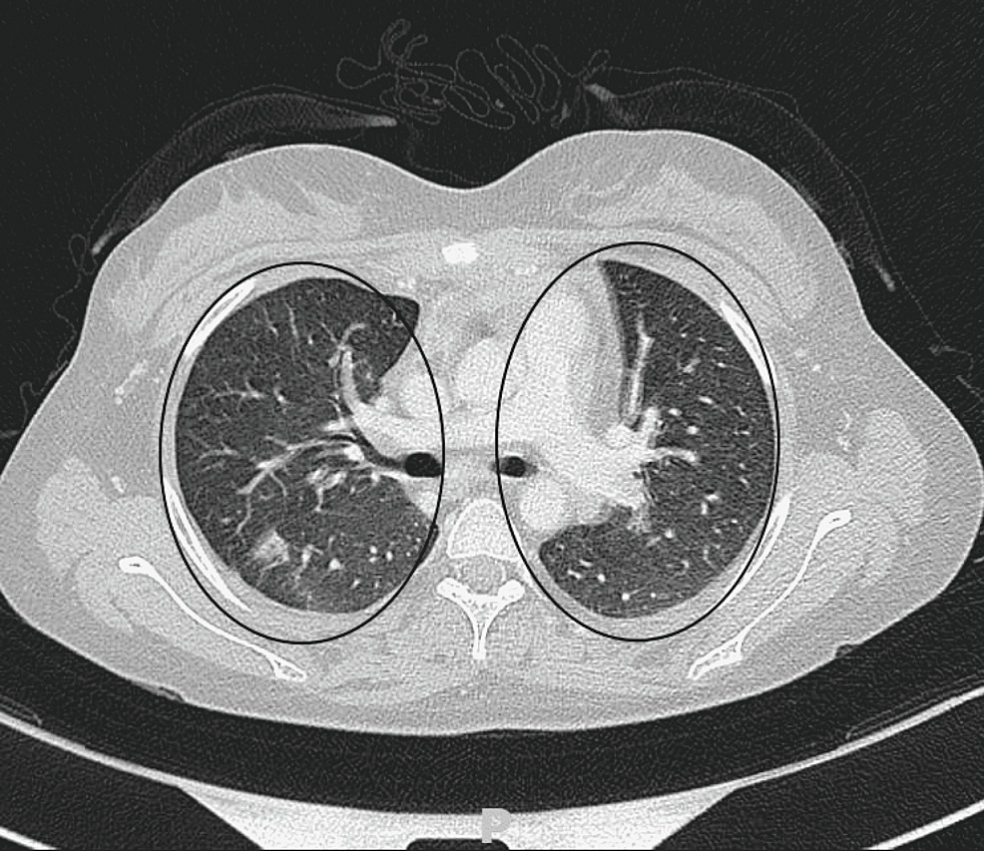
Chest scan image showing a significant regression of intra-parenchymal condensation after chemotherapy.

A myelogram was then performed to check for bone marrow involvement and showed no blasts or lymphomatous cells. Therefore, the decision was to put the patient on vincristine, etoposide, prednisone, and doxorubicin (OEPA) and cyclophosphamide, vincristine, prednisone, and dacarbazine (COPDAC) (DH-MA 2012 protocol). After two cycles of OEPA and four cycles of COPDAC, the patient reported no complaints and showed a favorable clinical evolution. The follow-up chest scan showed significant regression of intra-parenchymal condensation (Figure 4).

## Discussion

CHL is a malignancy of the lymphoid tissue characterized histologically by the presence of large binucleated or multinucleated neoplastic cells termed Hodgkin and Reed/Sternberg (HRS) cells against a background of benign inflammatory cells expressing CD30 [3]. However, nodular lymphocytes, predominantly HL, are characterized by the absence of HRS cells and expression for CD20 while being negative for CD30 [3]. Of note, CHL accounts for the majority of pediatric HL cases [3].

HL is rare in children under five years of age, with a prevalence of 10% to 12% in children ≤ 14 years of age with a reported male predominance [5]. However, it is the most commonly diagnosed type of cancer in adolescents between the ages of 15 and 19 [5]. Increasing family size, lower socioeconomic status, and early exposure to Epstein-Barr virus (EBV) are commonly cited risk factors in children [5]. Among adolescents and young adults HL (AYA HL), risk factors may include early birth order, higher socio-economic status, and delayed exposure to EBV [5].

HL can vary in severity; it falls in a spectrum from the coincidental discovery of mediastinal lymphadenopathy to life-threatening airway obstruction or spinal cord compression [6]. Due to the indolent onset of symptoms, most patients appear asymptomatic or with less noticeable symptoms [6]. The most common clinical feature of pediatric HL is painless lymphadenopathy in the cervical or supraclavicular region [6]. Lymphadenopathy is usually firm, elastic, and painless, but may be tender if it has grown rapidly. As the disease progresses, abnormal nodes create large nodal masses that fix to underlying tissue [6]. A third of patients have systemic symptoms such as fever, night sweats, and weight loss; many have persistent pruritus. Intrathoracic HL may be associated with nonproductive cough, dyspnea, chest pain, or superior vena cava syndrome [6]

Unlike cervical lymphadenopathy, which is commonly present in association with infectious and inflammatory diseases, supraclavicular lymphadenopathy should prompt earlier evaluation of a malignant origin [6]. It is important to note that because reactive lymphadenopathy is so common in children, antibiotics are still frequently prescribed before lymph node biopsy [5,6]. In our case, the patient was admitted to another hospital where antibiotics were administered for suspicion of a pulmonary infectious origin but without improvement and she was referred to our hospital structure for further investigations. A unique feature in our case is the presence of axillary and inguinal lymphadenopathy, which is uncommon and represents less than 5% of pediatric cases with infra-diaphragmatic involvement [6].

According to a retrospective analysis of 281 lymphomas with different histologies, extranodal involvement is more likely in non-Hodgkin's lymphoma (NHL) than in HL [7]. In addition, involvement of the lung parenchyma is uncommon in HL, with the most common finding in secondary being a mass-like consolidation greater than 1 cm with or without cavitation [7]. To the best of our knowledge, no similar case was reported on pediatric HL revealed by cannonball feature. However, although not concerning the pediatric population, a case report similar to ours, authored by Ingles et al., described a cannonball appearance revealing a HL in a 22-year-old patient [7].

A biopsy is mandatory to diagnose LH. Since the architecture of the lymph node is particularly critical for a correct diagnosis, fine-needle aspiration or central-needle biopsies are inadequate [8]. The malignant Reed-Sternberg cell must be identified in the cellular context of normal reactive lymphocytes, eosinophils, and histiocytes are important elements to confirm the final diagnosis [8]. In our case, a biopsy of the axillary lymphadenopathy revealed the diagnosis of NSCHL, the most common subtype, most commonly affecting adolescents and young adults and presenting with localized disease involving the cervical, supraclavicular, and mediastinal regions concerned [8]. The American College of Chest Physicians recommends that in patients who are candidates for curative therapy, each nodule be evaluated separately and curative treatment not withheld unless there is histopathologic evidence of metastasis [9]. The positron emission tomography (PET) scan is now considered essential for the initial staging of HL and a great help in selecting the appropriate therapy and is often performed in conjunction with a CT scan [8]. Our main limitation was that the pulmonary masses themselves were not biopsied. Relying solely on the result of the biopsy of the adenopathy to confirm that the pulmonary nodules are also related to HL is not failproof. However, in our case, we retained the presumptive diagnosis of HL with secondary pulmonary localization according to the clinical context.

In terms of differential diagnoses, TB tops the list especially in an endemic setting like ours because they both share similar signs and symptoms, laboratory test results, and imaging procedures. Concomitant cases of TB and HL have been reported. However, the most specific and sensitive diagnostic technique remains biopsy with histological examination [10-12].

In patients with HL, combined modality therapy (CMT) has been shown to improve outcomes. Patients with early favorable stage HL are treated with six cycles of chemotherapy alone or with CMT, which includes two to four cycles of chemotherapy and RT [13]. Two cycles of doxorubicin, bleomycin, vinblastine, and dacarbazine (ABVD) chemotherapy followed by 20 Gy RT were shown to be equally efficacious as four cycles of ABVD followed by 30 Gy RT in these patients [13]. In early-stage unfavorable HL, it was found that two cycles of bleomycin, etoposide, doxorubicin, cyclophosphamide, vincristine, procarbazine, and prednisone (BEACOPP) and two cycles of ABVD followed by 30 Gy RT improved outcomes but not overall survival [13].

## Conclusions

HL remains the most common cancer in adolescents particularly with risk factors. Lung involvement is common, but the demonstration of peripheral lymphadenopathy, particularly at the cervical level, is the key to diagnosis. The clinician must be vigilant of the persistence of symptoms and consider including HL as a differential diagnosis, as was the case in our patient who was admitted with suspected pulmonary infection after receiving antibiotic therapy. To date, no case of HL manifesting by a radiological appearance of "cannonball" has been reported in the medical literature. The final diagnosis is based on histopathological examination. The prognosis is excellent with a great curability.
